# Exploring the clinical features of minimally verbal autistic children

**DOI:** 10.3389/fpsyt.2025.1549092

**Published:** 2025-03-20

**Authors:** Silvia Guerrera, Elisa Fucà, Emanuela Petrolo, Andrea De Stefano, Laura Casula, Maria Grazia Logrieco, Giovanni Valeri, Stefano Vicari

**Affiliations:** ^1^ Child Adolescent Neuropsychiatry Unit, Bambino Gesù Children’s Hospital IRCCS, Rome, Italy; ^2^ Child Neurology and Psychiatry Unit, Fondazione Policlinico Universitario Agostino Gemelli IRCCS, Rome, Italy; ^3^ Department of Neuroscience, Catholic University of the Sacred Heart, Rome, Italy; ^4^ Department of Humanities, University of Foggia, Foggia, Italy; ^5^ Life Sciences and Public Health Department, Catholic University, Rome, Italy

**Keywords:** verbal language, psychiatric co-occurrences, behavioral symptoms, intellectual functioning, parenting stress, communication difficulties

## Abstract

**Introduction:**

It is recognized that around 25-30% of autistic children do not develop functional speech and remain minimally verbal beyond the age of 5. However, little is known about the clinical characteristics of this group.

**Methods:**

We retrospectively examined a sample of 189 autistic children and adolescents classified as minimally verbal (mean age: 7.37 ± 1.51; 152 males, 37 females) and compared them with a group of 184 verbal autistic children and adolescents (mean age: 7.71 ± 2.52; 160 males, 24 females). We considered intellectual functioning, severity of autism, emotional and behavioural problems, and parenting stress.

**Results:**

Children in the minimally verbal group exhibited significantly lower nonverbal Intelligent Quotient and an increase in restricted repetitive behaviours compared to the verbal group. Exploring potential differences in emotional and behavioural problems, the verbally group showed higher levels of anxiety symptoms. In addition, minimally verbal group showed high score of parenting stress.

**Discussion:**

This study highlights the importance of accurately characterizing minimally verbal autistic children and adolescents to facilitate the identification of specific and individualized interventions based on individual functioning profiles.

## 1 Introduction

According to the Diagnostic and Statistical Manual of Mental Disorders, 5th Edition, Text Revision (DSM-5-TR), Autism Spectrum Disorder (ASD) is defined as a neurodevelopmental condition. It is characterized by deficits in social communication and interaction, as well as repetitive and restricted patterns of behavior and interests (e.g., repetitive body movements such as hand flapping, sensory sensitivities, and circumscribed interests) (1). The male sex is widely recognized as one of the most established etiological factors for ASD, with an estimated prevalence of 3.8 times higher among boys than girls (2), leading to the concept of a “female protective effect,” where females may require a greater etiological burden to exhibit the same level of impact as males. The current global prevalence of ASD is estimated to be approximately 1% (3) and has significantly increased over the last 20 years (4).

About 25-30% of autistic children do not develop or fail to develop functional spoken language and remain *minimally verbal* past age 5 (5, 6), with implications for social and adaptive functioning in adulthood (7). The recent literature has attempted to delve into the variables that may influence the different levels of language development in autistic children (8). As previously noted by Chenausky (9), language development is closely linked to speech development, and a challenge remains in understanding how these two aspects may intertwine in children with a neurodevelopmental disorder such as ASD. The term “minimally verbal” (MV) usually refers to children who exhibit a limited vocabulary consisting of a small number of spoken words and fixed phrases (7).

Using a broader definition, Chakrabarti and colleagues, in 2017, defined MV children whose communicative abilities are severely limited due to a language deficit, a language disorder, intellectual disability, deficits in social and cognitive skills, or a combination of these factors. According to Kasari et al. (10), MV autistic children are characterized by “a highly restricted repertoire of spoken words or fixed phrases used for communication”; additionally, echolalic or stereotyped language may be present. The specific number of words used to define MV autistic children and the criteria for determining this may vary, ranging from 1 to 10 words (11), 25 or fewer words (12). More recently, Chenausky and collaborators have referred to a range of fewer than 20 intelligible words (13). Similarly, methods for clinical evaluation may be based on the number of different words in a natural language sample or on clinical judgment. In 2015, Norrelgen and colleagues (6) defined MV autistic children as those who, according to the Vineland Adaptive Behavior Scales (14), had a language age of less than 24 months and used between three words and some combinations of two words. In 2016, a study conducted by Plesa-Skwerer and colleagues defined MV autistic children as those who, according to parental reports, did not use phrase speech spontaneously and effectively and/or produced fewer than 30 words (15). The use of standardized tests commonly employed in language therapy to evaluate the language skills of MV autistic children has been questioned due to the complexity of instructions and the need for active participation from the child (16). Therefore, a common definition of MV autistic children is associated with the use of the Autism Diagnostic Observation Schedule, Second Edition (ADOS-2) (17), where “minimally verbal” refers to individuals assessed with Module 1 of the ADOS-2, designated for children aged over 30 months who have no speech or use very few words in simple combinations (17–21).

Investigations into intellectual functioning in this population have yielded highly heterogeneous results (18). General intellectual disability has been reported in MV autistic individuals (22–24). Additionally, it has been reported that, in MV autistic preschoolers, nonverbal intelligence quotient is one of the major predictors of subsequent language gains for those children who acquire some language before the age of 5 (20, 25–27). On the other hand, Bal and colleagues (18) reported that 16% of children in their sample had non-verbal cognitive abilities within average limits, contrasting with the hypothesis that minimal verbalization is synonymous with cognitive impairment. Additionally, Slusna and collaborators, studying a group of MV autistic youth and adults across the lifespan, found that 10.2% of the youth had a non-verbal intelligent quotient ≥ 70 (24).These results suggest that the mechanisms underlying the lack of language development are not solely attributable to cognitive level, but rather to a heterogeneous set of predictive factors, some of which are precursors to language that vary across the autism spectrum (26, 28).

Regarding the severity of ASD, previous research has suggested that MV autistic children do not exhibit significantly higher scores than verbal individuals (29). In a longitudinal study examining a group of MV preschoolers, none of the ADOS Calibrated Severity Scores (CSS) were correlated with the subsequent development of phrase speech (20). It remains to be determined whether there is a possible association between ASD core symptom severity and language abilities, given that most observational measures of ASD symptom severity are heavily influenced by language level (5, 16).

Previous research has described a higher prevalence of psychiatric comorbidities and emotional and behavioral problems in autistic children and adolescents (30–35). However, few studies have explored the relationship between emotional and behavioral problems in children with different language abilities (19). Indeed, it has been suggested that communication impairments can hinder development across various domains, with 25% of MV autistic children experiencing increased social withdrawal during adolescence (36). Moreover, MV autistic children are more likely to have associated oral-motor difficulties (37), and due to limited social interaction, adaptive behavioral skills, academic achievement, vocational success, and social relationships would also be affected (27, 38). Finally, it is necessary to consider that the inability to develop expressive verbal communication is a primary concern frequently reported by parents of autistic children (39). Parents of autistic children typically encounter higher levels of parenting stress compared to parents of children with other disabilities (40). Recently, it has been highlighted that children’s linguistic and communication difficulties appear to be a common source of parental stress and valid predictors of it (41). However, these difficulties have often been associated with the presence of greater emotional and behavioral problems (42), leaving the directly involved causes unexplored.

Considering that the current research has primarily focused on autistic individuals characterized as “high functioning” or having “lower symptom presentation”, the subgroup of ASD children who are MV is significantly underrepresented in both descriptive and intervention studies (13). In 2017, the strategic plan of the Interagency Autism Coordinating Committee underscored the imperative need to study autistic children exhibiting extremely limited verbal abilities, with the aim that 90% of autistic children acquire functional speech by age 5. Of note, most studies on MV autistic children and adolescents have explored small sample sizes (43). Therefore, the present study aimed to compare the clinical characteristics of a large group of MV autistic children and adolescents with a group of age- and sex-matched verbal autistic children and adolescents, specifically exploring possible differences in terms of intellectual functioning, autistic symptomatology and associated emotional and behavioral problems. Finally, maternal stress levels in the two populations were investigated.

A better understanding of the functioning characteristics of this group of autistic children can facilitate the development of personalized diagnostic strategies and identify specific interventions developed based on a child’s interests and strengths in other areas.

## 2 Materials and methods

### 2.1 Procedure and participants

Data were retrospectively collected from an in-depth review of the files of patients who referred to the Child and Adolescent Neuropsychiatry Unit of a third level Children’s Hospital between 2017 and 2023 for a neuropsychiatric evaluation following pediatrician’s clinical suspicion of ASD or for clinical follow-ups after receiving ASD diagnosis. Routine assessment procedure always included neuropsychiatric examination, cognitive and adaptive functioning evaluation, assessment of ASD symptoms and an emotional and behavioral evaluation. Grouping was based on the ADOS–2 module completed with the child (minimally verbal = Module 1, based on the use of module 1 designed for children with an absence of language or with production of a few single words; verbal = Module 2 or 3). Inclusion criteria were as follows: ascertained diagnosis of ASD supported by gold-standard instruments; age comprised between 5 and 18 years. Exclusion criteria were as follows: presence of neurological conditions (e.g., epilepsy); presence of genetic syndromes. The study was conducted according to the guidelines of the Declaration of Helsinki and approved by the local Ethics Committee (protocol code: 2423_OPBG_2021, approved on 27 October 2021).

The sample included 189 autistic children and adolescents classified as MV (MV group) and 184 age- and sex-matched verbally autistic children and adolescents (VB group) aged 5-18 years. **Tables 1**, **2** summarize the demographic characteristics of the sample and the ADOS-2 data (modules and scores), respectively.

**Table 1:** Demographic characteristics of the sample * *p* value <0.05.

	MV N=189	VB N=184	*p*
**Age**	7.37 ± 1.51	7.71 ± 2.53	0.114
**Sex (M/F)**	152/37	160/24	0.09
**Non-verbal intelligent quotient (range)**	59.59 ± 16.55	87.6 ± 16.07	<0.001*

**Table 2:** Autism Diagnostic Observation Schedule, Second Edition (ADOS-2) data for each group.

	MV N=189	VB N=184
**ADOS-2 Module 1 (%)**	100%	0%
**ADOS-2 Module 2 (%)**	0%	47%
**ADOS-2 Module 3 (%)**	0%	53%
**ADOS-2 SA (CSS)**	6.46 ± 1.62	5.98 ± 1.55
**ADOS-2 RRB (CSS)**	7.43 ± 1.43	6.47 ± 1.95
**ADOS-2 Total Score (CSS)**	6.89 ± 1.24	5.85 ± 1.46

### 2.2 Measures

#### 2.2.1 Autistic symptoms assessment

The diagnosis of ASD was established in accordance with the DSM-5 and was confirmed by the administration of the “gold-standard” instruments for the assessment of ASD symptoms, namely the Autism Diagnostic Observation Schedule, 2nd Edition (ADOS-2) (17) and the Autism Diagnostic Interview-Revised (ADI-R) (44). The ADOS-2 is a semi-structured direct assessment of communication, social interaction, and play or imaginative use of materials for individuals with a suspected diagnosis of ASD. The ADOS-2 consists of five modules designed for children and adults with different levels of language, ranging from nonverbal to verbal; it was administered and scored by licensed clinicians. Total score combines symptoms from the Social Affect (SA) and Restricted and Repetitive Behaviors (RRB) domains. In the analyses, the CSS were considered for the ADOS-2. The ADI-R is a standardized, semi‐structured interview during which caregivers report information about an individual suspected of having an ASD. The instrument generates algorithm scores for each of the three subdomains of autistic symptoms: qualitative impairments in reciprocal social behavior; qualitative abnormalities in communication and restricted range of interests and/or stereotypic behaviors.

#### 2.2.2 Cognitive assessment

Cognitive development was assessed by the Leiter International Performance Scale – 3rd Edition - Leiter-3 (45) – which provides a nonverbal measure of intelligence and assesses the ability to reason by analogy, by matching and perceptual reasoning in general, irrespective of language and formal schooling. The Global Non-Verbal Intelligent Quotient obtained through this test is based on four subtests: Figure Ground, Form Completion, Classification and Analogies, and Sequential Order. We used also the Colored Progressive Matrices, a 60-item test to assess mental ability associated with abstract reasoning, and considered a nonverbal estimate of fluid intelligence. The test consists of increasingly difficult pattern matching tasks and has little dependency on language abilities. All participants (100%) in the MV group were assessed using the Leiter-3, while 11% of the VB group were assessed using the Colored Progressive Matrices.

#### 2.2.3 Behavioral and psychological screening

Child Behavior Checklist (CBCL). Behavioral and psychological screening was performed by means of the parent-report questionnaire CBCL (46). For preschoolers, we used the CBCL for ages 1.5 to 5, which consists of 100 problem items. The instrument generates seven syndrome scales and five DSM-oriented scale profiles, consistent with the diagnostic categories of DSM-IV-TR and DSM-5. For participants aged 6-18 years, we used the CBCL 6-18, which generates eight syndrome and embraces six DSM-Oriented scales. Both versions include three general domains, namely internalizing, externalizing and total problems. In the current study, we considered the three general domains and the DSM-Oriented scales overlapping in the two versions of the instrument (i.e., versions for ages 1.5-5 and 6-18 years), namely Affective problems, Anxiety problems, Attention-Deficit/Hyperactivity problems, and Oppositional defiant problems. Clinical Cutoffs Description: T-Scores: 65-69 (Borderline) and 70+ (Clinical) are generated for narrow band scales. Moreover, for each participant we calculated the Dysregulation Profile (DP) of CBCL as described elsewhere (47, 48). Briefly, CBCL-DP is characterized by simultaneous high values in three syndrome scales, namely anxious/depressed, attention problems, and aggressive behavior, using the criteria based on a sum of t-scores, deficient emotional self-regulation as an aggregate cut-off score of >180 but <210 (elevation of 1 Standard Deviation - SD) on the abovementioned scales and Severe Dysregulation as an aggregate cut-off score of ≥210 (elevation of 2 SD or more). Of note, data from CBCL were available for 142 out of 189 participants in the MV group.

#### 2.2.4 Maternal stress assessment

To investigate maternal stress levels, the Parenting Stress Index-Short Form (PSI) (49) was used. PSI is an easy-to administer tool to measure maternal stress. It consists of 36 questions and each item is rated on a 5-point Likert scale from (1) strongly disagree to (5) strongly agree. The PSI captures three domains—parental distress (PD), parent–child dysfunctional interaction (P-CDI), and difficult child (DC). The sum of all questions results in the Total Stress score. PSI has been translated into several languages and has been frequently used in ASD research (50–53). Data from PSI were available for a subgroup of 262 mothers (N= 148 for VB group and N=114 for MV group).

### 2.3 Statistical analysis

Descriptive statistics were used to analyze demographic and clinical characteristics of the whole sample. Chi-squared test was used to investigate group differences on sex distribution. Group differences were examined by t test and analysis of co-variance (ANCOVA), adjusting for IQ. *Post hoc* analyses were performed using Tukey HSD test. Partial eta squared (ηp^2^) was used to measure effect size. A p-value less than or equal to 0.05 was considered as statistically significant.

## 3 Results

### 3.1 Differences on cognitive level and autistic symptoms

Children belonging to the MV group exhibited significantly lower IQ than children in the VB group (59.59 ± 16.55 and 87.6 ± 16.07, respectively). The 26.5% of participants in the MV group exhibited a non-verbal intelligent quotient ≥ 70 compared to 90% of participants in the VB group. Regarding the investigation of potential group differences on ASD symptoms, measured by ADOS-2 CSS, ANCOVA (IQ as covariate) failed to detect significant differences on the SA domain F (1,357) =.462, p = 0.497, ηp^2^ = 0.01; 6.46 ± 1.62 and 5.98 ± 1.55 for MV and VB, respectively). On the other hand, participants in the MV groups exhibited significantly higher scores on the Restricted and RRB domain F(1,357) = 4.372, *p* = 0.037, ηp^2^ = 0.012; 7.43 ± 1.43 and 6.47 ± 1.95, respectively).

### 3.2 Differences on CBCL scores

Multivariate ANCOVA showed that participants in the MV exhibited significantly lower scores than participants in the VB group in the anxiety DSM-Oriented scale of the CBCL. **Table 3** summarizes the results. No group differences emerged in the CBCL DP scores (180.59 ± 19.99 and 183.4 ± 21.86 for MV and VB groups, respectively; *p* = 0.228).

**Table 3:** Group differences on Child Behaviour Checklist (CBCL) scores.

	MV N=142	VB N=184	*p*	η_p_^2^
**Internalizing problems**	61.06 ± 9.9	61.88 ± 9.9	0.757	<0.000
**Externalizing problems**	57.28 ± 9.45	56.69 ± 9.3	0.062	0.009
**Total Problems**	62.55 ± 9.7	61.74 ± 9.66	0.509	0.001
**Affective problems**	62.24 ± 8.7	61.85 ± 9.5	0.761	<0.000
**Anxiety problems**	59.64 ± 8.64	62.9 ± 7.95	0.003*	0.028
**Attention-Deficit/Hyperactivity problems**	59.78 ± 6.5	60.49 ± 7.6	0.696	<0.000
**Oppositional defiant problems**	55.02 ± 6	56.54 ± 6.39	0.404	0.002

### 3.3 Differences on maternal parenting stress levels

ANCOVA (IQ as covariate) failed to detect significant differences on the PD scale of the PSI F(1,259) =1.167, *p* = 0.28, ηp^2^ = 0.004; 29.52 ± 10.11 and 26.88 ± 10.42 for MV and VB, respectively), as well as in the DC scale F(1,259) =.010, *p* = 0.919, ηp^2^ < 0.000; 31.07 ± 10 and 31.8 ± 15.18 for MV and VB, respectively). On the other hand, mothers of participants in the MV groups exhibited significantly higher scores on the P-CDI scale F(1,259) = 5.213, *p* = 0.023, ηp^2^ = 0.019; 26.68 ± 7.14 and 24.13 ± 7.3, respectively).

## 4 Discussion

The main goal of this study was to provide a description of the clinical characteristics of a large group of autistic children, defined as MV based on the use of module 1 of the ADOS-2 and compared to a sex- and age-matched group of verbal autistic children. Participants in the MV group showed below-average intellectual functioning compared to the VB group, with a relatively small subgroup showing a non-verbal intelligent quotient falling in the normal range. Several studies have highlighted the significant variability in skills across various domains of cognitive, social, and linguistic functioning among autistic individuals. The prevalence of intellectual disability in autistic children with an average age of 8 years, calculated in the United States, has been estimated to be one-third (54). Although it is a common assumption that MV autistic individuals have a greater impairment in cognitive functioning (7, 9), recent studies have shown that some MV autistic children appear to report a typical or borderline non-verbal intelligent quotient (18, 24, 55), suggesting a fundamental divergence between verbal and nonverbal functioning in this population. This refutes the notion that reduced language abilities necessarily correspond to compromised intellectual abilities and, instead, suggests a wide range of profiles wherein language may be impaired or even absent in ASD children with otherwise intact intellectual abilities. Chenausky et al. (55) reported that the average non-verbal intelligent quotient of a sample of 54 MV autistic children was 68, with half of the sample having non-verbal intelligent quotient scores below 70. Slusna et al. (24) exploring 49 autistic individuals above 6 years of age minimally or nonverbal, using Leiter International Performance Test-Revised (Leiter-R), found a non-verbal intelligent quotient > 70, only in 5 participant. Finally, Bal et al. in 2016 (18), in a group of 257 MV autistic children and adolescents, 4–17 years old found a typical non-verbal intelligent quotient in 16% of individuals, with differences dependent on the individual assessment instruments used. In general, the rates of intellectual disability in the autistic population MV seem to vary depending on the IQ assessment used, the characteristic of sample explored (eg. age range) as well as the definition of MV identified. The limited number of studies including verbal autistic children with intellectual disability (56) and MV autistic children has not yet allowed for a clear understanding of the possible “double dissociation” between linguistic and intellectual functioning, as previously stated by Smith N & Tsimpli IM (57), who stated the independence of language from other forms of cognition. Further longitudinal studies are needed on this matter.

The second finding of the study showed that, regarding autistic symptomatology, the MV group exhibited a slight but significant increase in CSS scores in the RRB domain compared to the VB group, after accounting for IQ differences. Literature on the relationship between RRBs and language development remains contradictory. Bal and colleagues (18), as well as Zheng and collaborators (58), reported slightly higher RRB scores in children with “Few to No Words” compared to those with “Some Words”, based on ADOS Module 1, suggesting the existence of different levels of ASD symptom severity based on spoken language levels. Harrop and collaborators (59) exploring a group of MV autistic children (defined as children with fewer than 20 spontaneous functional spoken words) ages 5 to 8 years, reported high rates of verbal RRBs, assessed through observational coding of caregiver-child interactions, videotaped. On the other hand, our result differs from few studies that have investigated the association between autistic symptoms and verbal language development, where participants in the MV group do not exhibit significantly higher ADOS scores than verbal individuals (15, 20). In particular, Thurm and collaborators conducted a longitudinal study investigating language outcomes in a group of MV autistic children assessed during the preschool years. The authors showed that the severity of ASD symptoms in core domains, as indicated by the CSS score, did not predict the emergence of spoken language by the age of 5 (20). Furthermore, the variations in SA-CSS scores were no longer significant when nonverbal cognitive abilities were considered in the model. According to the authors, this result suggests the presence of collinearity between nonverbal cognitive abilities and the SA-CSS, which undermines the ability to accurately assess predictive models of language acquisition in ASD. This implication may indicate that, in the current study, the slight significance of the RRB scores in MV autistic children could be attributed to the higher prevalence of intellectual disability, compared to the VB group, which in some studies has been linked to a greater number of motor stereotypies included in RRB, particularly among older children (60). Beyond the current difficulty in providing unequivocal conclusions regarding this correlation (61), the possible presence of more severe autistic symptoms implies the need to provide alternative communication interventions. Our results, by providing a more appropriate understanding of certain clinical characteristics of MV autistic individuals, could support the development of specific interventions by leveraging certain peculiarities. As suggested by Harrop and colleagues (59), more severe autistic symptomatology in terms of RRBs, particularly those involving objects manipulation, could be leveraged within social communication intervention programs to promote greater child engagement. The focus would be on expanding or redirecting these behaviours, ultimately aiming for increased adherence to the communicative intervention.

An additional objective of the current study was to compare the MV group with the VB group on a measure for behavioural and psychological screening. In fact, few studies have explored how emotional and behavioural problems varies in children with different language abilities (19). Our results showed greater parent-reported anxiety issues in VB group compared to MV autistic children. The CBCL is one of the most widely investigated instruments to detect emotional and behavioural problems in children and adolescents with neurodevelopmental disorder (30), such as ASD. Many studies support its reliability and validity across different clinical groups (62). Data on the distribution of anxiety disorders in autistic children remain unclear, with some studies exploring the possible association with intellectual functioning and others with language functioning. Indeed, it is possible that certain psychiatric comorbidities (e.g., anxiety, depression) manifest differently in individuals with varying intellectual and language abilities (63). Relatively to cognitive functioning, some studies reported that low IQ may be associated with more anxious symptoms in ASD children (64–66). More recently, other studies, including a meta-analysis conducted on 49 papers on the topic, suggested that anxiety symptoms are more frequently seen among autistic children with borderline intellectual functioning, whereas those with intellectual disability generally show considerably lower anxiety symptom scores (67, 68). The difficulty in assessing anxious symptoms in autistic individuals due to their communication difficulties (69), high methodological variability in assessment (70), and the absence of good specific measures for this group of children (71) would explain the high variability in results, especially in the presence of a prevalence of intellectual disability (67). Additionally, it is necessary to consider that the lower levels of anxiety reported by parents of MV autistic children could be explained by the parents’ difficulty in recognizing anxious symptoms in their children, mainly due to reduced use of verbal language and anxiety-related behaviours overlapping with other behaviours (e.g., ASD characteristics), as suggested by Tarver and colleagues in 2021 (72). Indeed, most studies on anxiety in autistic children rely on parent reports (71). Depending on parental reporting can be challenging, as it often requires the child to effectively communicate their emotions to the caregiver. Consequently, psychiatric conditions such as anxiety may be underdiagnosed in autistic individuals with MV and intellectual disability. The limited communication skills in MV children, often associated with impaired intellectual functioning, may lead to significant difficulties in verbally expressing their concerns or identifying complex internal states such as anxiety or other internalizing symptoms. In this regard, Plesa-Skwerer and colleagues (73), examining a group of MV autistic children and adolescents in terms of psychiatric comorbidity and emotional dysregulation through the exclusive use of parent reports, reported low rates of emotional dysregulation despite a high percentage of psychiatric comorbidity. In this case as well, the authors attribute this result to the objective difficulty that parents have in understanding the internal states of minimally verbal children, as well as the persistent phenomenon of diagnostic overshadowing (34). Furthermore, consistently with the results of our study, no difference was found on the externalizing symptoms scales. Therefore, it would be useful for future studies to focus on examining the characteristics of the MV population, exploring their intellectual functioning in greater depth and how this may influence other aspects, including emotional and behavioral problems.

Finally, when exploring parenting stress in mothers of MV autistic children compared to parents of verbally autistic children, parents of MV group showed greater problems on the parent-child interaction subscale. It is acknowledged that parents of autistic children report higher levels of stress compared to parents of non-autistic children and children with other developmental disabilities (74–77). Although stress levels may vary across the different studies considered, depending on the composition of the sample and the tools used to measure stress, our findings appear to be in line with the literature on families of children with developmental disabilities, which has showed that the severity of child’s impairments seems to be an important factor related to parenting stress (78). Specifically, in families with autistic children, the elements of stress reported by the families have been found to be correlated with the degree of child’s impairment, including the severity of cognitive functioning (53, 74) and language functioning (79). In particular, PSI in parents of autistic children has often been linked to the child’s level of intellectual ability, as the latter is correlated with their autonomy, learning potential, communication skills, and the manifestation of problematic behaviors. In this regard, Scibelli and colleagues (53) found an association between cognitive impairment and PSI levels while exploring samples of autistic adolescents. From this, we could infer that our results on PSI levels in school-age children and MV adolescents might be associated not only with the communication difficulties of this population but also with the effect of intellectual impairment. In autistic children, additional factors that seem to affect parental stress are emotional and behavioural issues (80). Therefore, the increased stress in parents of MV autistic children could be related to the influence of all these factors.

Our study, which focused on a large sample of MV autistic children and adolescents, revealed a distinct profile for this population, characterized by lower cognitive functioning and slightly greater repetitive behaviours/restricted interests compared to the verbal group. From a psychopathological perspective, differences in anxiety symptoms between the two groups indicated a worse profile for verbal children and adolescents. Additionally, the MV group showed higher levels of parental stress compared to the ASD group with language abilities. Based on these findings, we emphasize the need for further research, particularly longitudinal studies, to address these challenges and develop tailored rehabilitative interventions for this diverse clinical population as early as possible.

This study has some limitations that must be taken into account: first and foremost, the retrospective nature of the study. Specifically, this determined the possibility of retrieving language assessment data for only 32% of autistic participants classified as MV. A linguistic assessment of both language production and comprehension in these children would have expanded the understanding of this sample. Second, our sample was defined as MV based on one of the definitions considered in the literature, in terms of linguistic function and age, but since there is still no established operational definition for MV in autistic individuals, the results of the current study are not fully comparable to the results of previous studies. A similar consideration must be made regarding the assessment tools used to define the sample, although we used Module 1 of the ADOS, which falls among the instruments considered in the literature as valid for one of the definitions. Third, a notable limitation of this study is the age range included in both the verbally fluent and minimally verbal groups, which spans from 5 to 18 years. Although the age range was chosen to ensure a broad representation of individuals, it is important to acknowledge that the linguistic capabilities of children at the upper end of this spectrum, especially in the verbally fluent group, may differ significantly from those at the lower end, or from those in the minimally verbal group. Thus, caution is necessary when drawing conclusions about language abilities across the age span, as the developmental trajectory of language acquisition may not be fully captured within these ranges. Future studies with more closely matched age groups or a more narrow focus on specific developmental stages may help to further refine our understanding of these differences In addition, although the CBCL is one of the most commonly used measures for behavioural and psychological screening in autistic children, it includes many items directly dependent on verbal language abilities, which may not be appropriate for ASD (81) and MV individuals.

Despite these limitations, given the limited representation of MV autistic children in both descriptive and intervention research (81), the current study explored a large sample of scholar MV autistic children and adolescents, contributing to the existing literature. The observed variability in cognitive and linguistic abilities among MV autistic children supports the idea that no single underlying mechanism explains why these children do not acquire speech. On the other hand, previous research has highlighted that a deeper understanding of the characteristics of non-verbal or minimally verbal autistic children—particularly in terms of intellectual and linguistic functioning, as well as multiple behavioral domains across various social contexts—could be achieved through their involvement in large-scale intervention studies. This, in turn, may enhance our comprehension of how these children respond to treatments, ultimately paving the way for the development of personalized interventions to optimize outcomes (7). Koegel and colleagues (82), in considering the factors that may facilitate the implementation of specific interventions, emphasize not only the importance of establishing a clear definition of MV based on word count and age, along with the development of precise and specific assessment tools, but also the need for studies estimating verbal and/or non-verbal cognitive abilities. By identifying the child’s overall functioning, such studies could be valuable for *post hoc* analyses of intervention effects in both nonverbal and MV individuals. In this regard, the findings from our sample may contribute to a deeper understanding of MV autistic individuals. Finally, regardless of the underlying causes of the heightened stress levels observed in the parents of this group of children and adolescents, this finding underscores the need for targeted interventions to provide them with support, particularly during the school years. While parent-mediated therapies have been designed for preschool children with ASD (83, 84), there are still limited programs addressing the needs of families of autistic adolescents (53), specially MV. As emphasized by Scibelli and colleagues, it is essential to develop intervention strategies that actively involve parents without adding to their caregiving responsibilities, such as home-based support or tools like video feedback to enhance their perception of interactions with their child. Future studies, including homogeneous age groupings (43), or including specific age groups as such as adolescence, may encourage stakeholders and decision-makers to improve their efforts to provide targeted interventions and support services. Furthermore, given the severe lack of research on language and communication interventions for MV autistic children aged 5 and above, future studies exploring communication development in MV autistic children are essential.

## Data Availability

The data that support the findings of this study are available on request from the corresponding author. The data are not publicly available due to privacy or ethical restrictions.
